# Mechanisms of Progranulin Action and Regulation in Genitourinary Cancers

**DOI:** 10.3389/fendo.2016.00100

**Published:** 2016-07-27

**Authors:** Ryuta Tanimoto, Kuojung G. Lu, Shi-Qiong Xu, Simone Buraschi, Antonino Belfiore, Renato V. Iozzo, Andrea Morrione

**Affiliations:** ^1^Biology of Prostate Cancer Program, Department of Urology, Kimmel Cancer Center, Thomas Jefferson University, Philadelphia, PA, USA; ^2^Cancer Cell Biology and Signaling Program, Department of Pathology, Anatomy and Cell Biology, Kimmel Cancer Center, Thomas Jefferson University, Philadelphia, PA, USA; ^3^Department of Health Sciences, Endocrinology, University Magna Graecia of Catanzaro, Catanzaro, Italy

**Keywords:** progranulin, bladder and prostate cancer, motility, anchorage-independent growth, tumor formation *in vivo*

## Abstract

The growth factor progranulin has emerged in recent years as a critical regulator of transformation in several cancer models, including breast cancer, glioblastomas, leukemias, and hepatocellular carcinomas. Several laboratories, including ours, have also demonstrated an important role of progranulin in several genitourinary cancers, including ovarian, endometrial, cervical, prostate, and bladder tumors, where progranulin acts as an autocrine growth factor thereby modulating motility and invasion of transformed cells. In this review, we will focus on the mechanisms of action and regulation of progranulin signaling in genitourinary cancers with a special emphasis on prostate and bladder tumors.

## Introduction

Progranulin, also known as proepithelin, granulin–epithelin precursor, acrogranin, and PC cell-derived growth factor, is a secreted glycoprotein with dual cellular roles, both as a growth factor regulating cell proliferation and wound repair and as a component of the transforming machinery in several cancer systems (1–3). Progranulin regulates inflammation and neurodegeneration (4) and has been linked to the development of frontotemporal dementia (FTD) (4).

Progranulin contains seven and a half granulin-like domains, which are ~6 kDa peptides called granulins (granulin A to G and paragranulin). These domains contain highly conserved tandem repeats of 12-cysteine residues, with the exception of granulin G, which has only 10 cysteine residues. The progranulin precursor is a 68.5-kDa protein of 593 amino acids, which migrates at ~88 kDa on Western immunoblot due to glycosylation (5).

Historically, granulins were first discovered as a novel class of leukocyte peptides with cytokine-like activities (6, 7). The progranulin precursor protein was later purified from PC cell-derived conditioned medium by Zhou et al. (8), who demonstrated the function of progranulin as mitogen and an autocrine growth factor for cancer cell lines. This precursor protein was identical to a growth-promoting factor later purified by Dr. Baserga’s group from the conditioned media of BRL-3A rat liver cells (8), which was able to induce cell proliferation of R^−^ cells, derived from mice with a targeted deletion of the insulin-like growth factor receptor (*IGF-IR*) gene (9). Significantly, progranulin was the only known growth factor able to bypass the requirement for the IGF-IR, thus promoting growth of R^−^ cells (10). As demonstrated by He et al., progranulin overexpression in adrenal carcinoma and renal epithelial cells resulted in enhanced progranulin secretion, increased mitogenesis, and tumorigenesis (11). Progranulin has been shown to enhance proliferation and promote tumor growth in several cancer cell lines, such as breast, gastrointestinal, hepatic, lung, and genitourinary cancers. In this manuscript, we review progranulin’s dual roles as a novel transforming growth factor and a clinical diagnostic and prognostic biomarker in genitourinary cancers.

## Progranulin in Ovarian Cancer

Ovarian cancer (OC) is the deadliest gynecological cancer and is the fourth leading cause of cancer-related deaths in women (12). While current treatment with surgery and chemotherapy can manage early stage disease, patients with late stage disease often recur and develop distant metastases. From the initial diagnosis, the median 10-year survival is only about 40% (13). Thus, it is very important to identify novel predictors of ovarian tumor progression.

Progranulin action in OC has been well characterized. Jones et al. (14) compared progranulin expression between low malignant potential and invasive ovarian tumors and demonstrated that progranulin expression was upregulated in invasive OC compared to low-grade tumors (15), suggesting that progranulin expression may be predictive of tumor progression. Progranulin mRNA and protein expression levels were significantly higher in tissue samples obtained from OC when compared to benign and normal ovarian tissues (15–17). Furthermore, progranulin expression level was correlated with higher stage and worse prognosis in OC patients (16–20).

Furthermore, numerous *in vitro* and *in vivo* studies have demonstrated the role of progranulin in promoting proliferation, resistance to apoptosis, and motility of OC cells, where progranulin activates mitogen-activated protein kinase (MAPK) and MMP-2 signaling (14, 17, 20–22).

Another potential serum marker for OC is serum leukocyte protease inhibitor (SLPI), a serine protease inhibitor protein, whose levels are elevated in serum derived from patients with OC (23). Significantly, SLPI protects progranulin from serine proteases and cleavage into mature granulins (24). Devoogdt et al. demonstrated that SLPI overexpression protected progranulin from elastase-mediated degradation and increased OC growth and survival, both *in vitro* and *in vivo* (25, 26). Han et al. reported that among progranulin, SLPI, and the FDA approved serum biomarkers HE4 and CA125, progranulin was the only biomarker independently associated with poor prognosis in OC patients in remission after initial surgical cytoreduction and chemotherapy (18).

## Progranulin in Cervical and Endometrial Cancer

Cervical cancer is the third most common cancer among women worldwide and the third most common genitourinary cancer in United States (12). Human papillomavirus (HPV) infection is the prevalent cause of cervical cancer as in fact over 99% of cervical cancers are positive for HPV (27). However, only a small fraction of women infected by HPV develops cervical cancer suggesting that other factors contribute to its progression.

Progranulin overexpression or recombinant progranulin protein stimulated transformation of HPV16-immortalized cervical mucosa epithelial H8 cells *in vitro* and *in vivo* (28, 29). On the contrary, inhibition of progranulin expression by siRNA approaches inhibited growth (28, 30).

Endometrial cancer is the most common genital cancer among women in United States, with an estimated 60,050 new cases in 2016 (12). The risk factors are related to an increase in circulating estrogens secondary to obesity, chronic anovulation and nulliparity estrogen replacement therapy, and tamoxifen use. Endometrial cancer tissues showed upregulated progranulin expression in association with estrogen receptor (31), and progranulin expression level was increased after estradiol and/or tamoxifen treatment *in vitro* (31). Similar to cervical cancer, inhibition of progranulin with shRNAs resulted in decreased endometrial cancer cell proliferation (31).

These results suggest that in endometrial cancer progranulin may work in estradiol-dependent fashion and mediate, at least in part, estrogen receptor-dependent biological responses. This is reminiscent of progranulin action in breast cancer, where progranulin (PCDGF/granulin precursor) expression is stimulated by estradiol (32) and progranulin regulates estrogen-dependent mitogenesis (33).

## Progranulin Action in Bladder Cancer

Bladder cancer is the fourth most common cancer in men in developed countries, with high recurrence rates of around 60% after treatment (12, 34, 35). The vast majority of early stages bladder tumors present as low-grade non-invasive papillary tumors (Ta stage). The remaining comprises tumors that have penetrated the basement membrane but not invaded the muscle layer of the bladder wall (T1 stage) and muscle-invasive tumors (T2–T4 stages) (36). The prognosis for low-grade tumors is generally good but about 15% of low-grade tumors progress to invasive disease. For invasive tumors, patient survival is ~50% at 5 years (37). Invasive tumors are frequently associated with metastases, which have a 5-year survival rate of 6% (37). However, despite the significant clinical impact of bladder cancer, there is still an urgent need to discover drivers of disease progression and novel therapeutic targets.

In recent years, our laboratories have established that progranulin plays a critical role in bladder cancer by regulating bladder cancer cell motility, invasion, anchorage-independent growth, and tumor formation *in vivo* (38–41).

We initially demonstrated that recombinant human progranulin did promote migration, wound closure, and invasion of urothelial cancer cells (42). Progranulin-mediated biological responses required the activation of the MAPK pathway and paxillin, which upon progranulin stimulation formed a complex with focal adhesion kinase (FAK) and active extracellular signal-regulated kinase (ERK) at the leading edge of migrating urothelial cancer cells (42). These results suggested that progranulin-induced cell motility might be regulated by the ability of progranulin to modulate focal adhesion dynamics of motile cancer cells.

We later discovered that urothelial cancer cells abundantly secrete progranulin (39). Significantly, stable depletion by shRNA approaches of endogenous progranulin expressed in T24 urothelial carcinoma-derived cell inhibited both the Akt and MAPK pathways, reduced the ability of T24 cells to proliferate in the absence of serum and inhibited motility, wound healing and invasion *in vitro* (39). Collectively, these results demonstrated that progranulin acts as a tumorigenic autocrine growth factor for bladder cancer cells and may work as a biomarker for bladder neoplasms.

**Figure 1:**
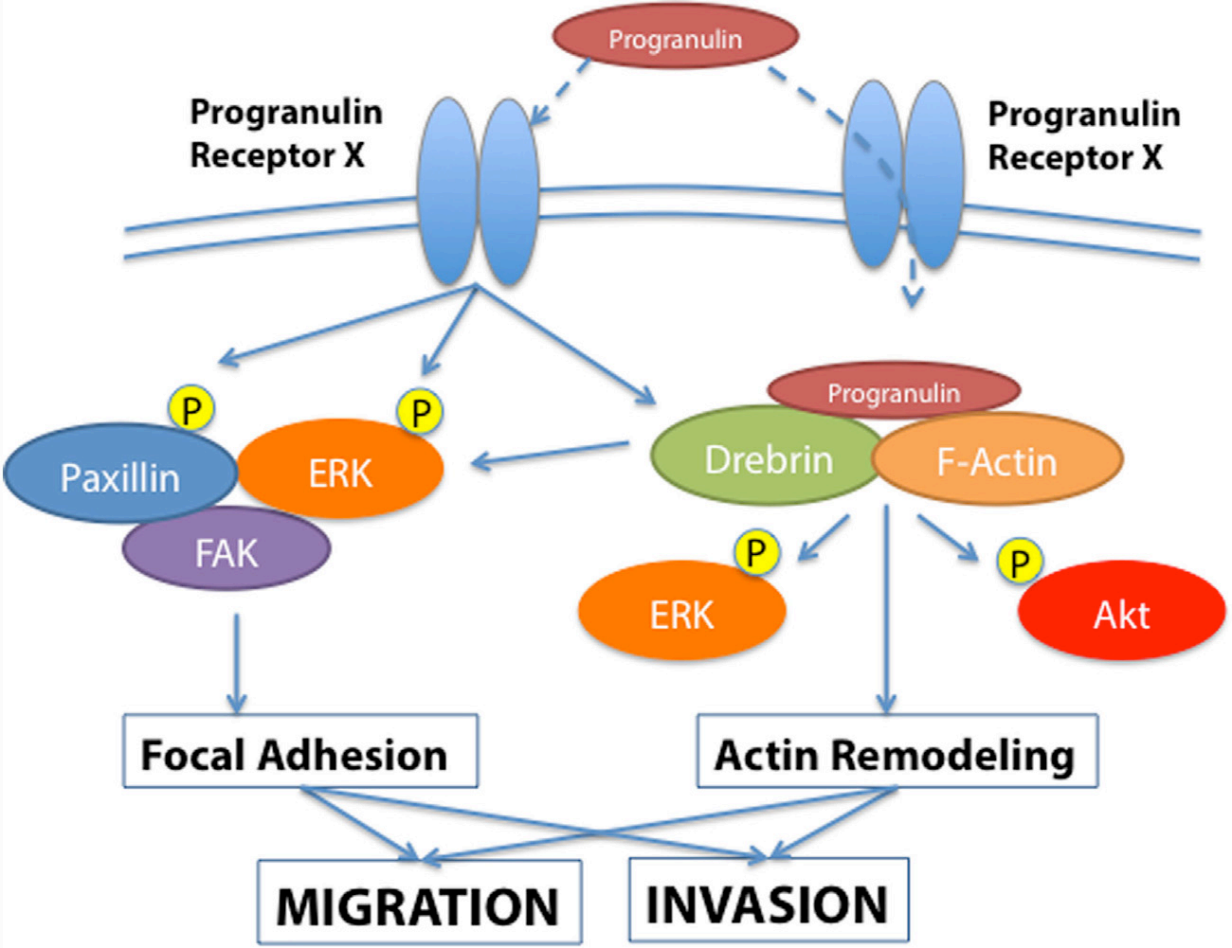
**Progranulin signaling in bladder cancer**. The schematic draw summarizes the current knowledge of progranulin signaling pathways leading to motility and invasion of bladder cancer cells. Progranulin interaction with the unidentified progranulin membrane receptor mediates progranulin internalization and interaction with the F-actin protein drebrin, thus regulating MAPK and Akt pathways and cytoskeletal remodeling (40). Additionally, progranulin signaling activates paxillin, which may contribute with MAPK in regulating the dynamic of focal adhesions.

In order to gain further insights into the molecular mechanisms of progranulin action in bladder cancer, we searched for novel progranulin-interacting proteins using pull-down assays with recombinant progranulin and proteomic analysis. We demonstrated that drebrin (*d*evelopmentally *re*gulated *br*ain prote*in*), an F-actin binding protein (43, 44), interacted with progranulin in urothelial cancer cells (40), modulated progranulin-induced MAPK and Akt signaling, and promoted progranulin-dependent motility and invasion by regulating F-actin remodeling (40). In addition, drebrin was essential for anchorage-independent growth *in vitro* and tumor formation *in vivo*, and its expression was upregulated in bladder cancer tissues compared to normal tissue controls (40). Our data demonstrated an essential functional role for drebrin in the regulation of progranulin actions suggesting that drebrin may constitute a novel target for therapeutic intervention in bladder tumors. In addition, drebrin may work as novel biomarker for bladder cancer progression (Figure 1).

Given the critical role of progranulin in regulating motility and invasion of urothelial cancer cells (39, 40, 42), we then determined whether targeting progranulin could have therapeutic efficacy in bladder cancer. Thus, we very recently generated progranulin-depleted tumorigenic urothelial cancer cells and demonstrated that progranulin targeting markedly reduced *in vivo* tumor growth of UMUC-3 cells in both orthotopic and subcutaneous xenograft tumor models (41). The immunostaining analysis of orthotopic tumors derived from progranulin-depleted UMUC-3 cells tumors had significantly lower expression level of Ki67, a cellular marker of proliferation, compared to tumor tissues derived from control-transfected cells (41). Moreover, the F-actin network of orthotopic tumors derived from progranulin-depleted cells was intact and well assembled compared to controls (41), supporting our previous *in vitro* results (40) and the hypothesis that progranulin might regulate invasion and motility of urothelial cancer cells by modulating F-actin remodeling.

Significantly, tumor tissues derived from UMUC-3/Control orthotopic xenografts showed reduced expression of the epithelial marker E-cadherin and increased levels of the mesenchymal protein vimentin compared to progranulin-depleted orthotopic xenografts. These results would suggest that the role of progranulin in bladder cancer tumor formation might be associated with an epithelial-to-mesenchymal transition (EMT) (45), which is severely affected by progranulin ablation in urothelial cancer cells. These results are consistent with data showing that in OC progranulin induces motility and invasion through EMT and the activation of cancer-associated fibroblasts (20).

The standard of care for invasive and metastatic bladder cancer is based on platinum-containing anti-cancer drugs, such as cisplatin (46). Progranulin depletion significantly enhanced cisplatin-dependent cell death as compared to control bladder cancer cells (41), suggesting that progranulin targeting in combination with cisplatin could enhance the therapeutic efficacy of cisplatin-based therapy in invasive bladder tumors.

Because progranulin might be secreted in the urine either through active secretion or after cell death, initially we assessed progranulin levels in concentrated urines of healthy subjects and demonstrated that progranulin can be detected in urine using immunoblots (39). Furthermore, progranulin levels in urine were quantified by enzyme-linked immunosorbent assay (ELISA) (39). Significantly, Soukup et al. (47) tested by ELISA assay the urinary concentrations of 27 biomarkers, including progranulin, for the detection of primary and recurrent urothelial bladder cancers. Importantly, the urinary concentration of progranulin was significantly higher in patients with bladder cancer compared to healthy subjects (47). However, progranulin levels did not show significant difference between patients with cancer recurrence and patient without recurrence (47). Taking into account the sample size limitation of this study, additional analyses on larger cohorts are necessary to establish whether progranulin may work as a urine biomarker for bladder cancer.

We recently analyze by immunohistochemistry progranulin expression levels in a human bladder tumor microarray containing 69 validated cases, including various types and stages of bladder cancers. Progranulin was barely detectable in normal bladder. In contrast, all malignant bladder tumors exhibited a significantly increased progranulin expression with the only exception of mucinous carcinoma where progranulin levels were comparable to normal tissues (41). Progranulin upregulation was not statistically different between low-grade and high-grade invasive urothelial carcinoma tissues (41). Progranulin was also upregulated in metastatic bladder tissues suggesting that progranulin expression levels might be associated with bladder cancer metastases. We additionally tested progranulin expression levels in archived paraffin-embedded tissues derived from non-invasive carcinoma *in situ* (Tis), non-invasive low-grade papillary carcinomas (Ta), non-invasive high-grade papillary carcinomas (Ta) using as control normal, and invasive high-grade carcinoma. Progranulin expression levels were significantly upregulated in non-invasive low-grade papillary carcinomas (Ta) compared to benign tissues and enhanced in both non-invasive high-grade papillary carcinomas (Ta) and high-grade carcinoma tissues compared to benign tissues, while in non-invasive carcinoma *in situ* (Tis) progranulin expression was at levels similar to benign tissues (41). These results were somewhat surprising considering the importance of progranulin in regulating motility and invasion and suggest that progranulin expression is predictive of bladder tumor formation but do not discriminate between superficial and invasive urothelial carcinomas. On the contrary, drebrin expression correlated with bladder tumor progression (40) indicating that expression levels of downstream progranulin effectors may be more predictive of bladder tumor progression than progranulin itself.

## Progranulin Action in Prostate Cancer

Prostate cancer is the most common cancer in men and is also the second leading cause of cancer-related deaths in men (12). In the developed world, most prostate cancers are diagnosed at an early stage where the cancer is confined to the prostate or local organs, with minimal risk of disease progression after treatment. However, a significant proportion of men with clinically localized prostate cancer treated with radical prostatectomy will develop biochemical recurrence, subsequent local recurrence, and distant metastases (48–50). Furthermore, the mechanisms promoting progression to the castration-resistant stage of prostate cancer (CRPC) are still very poorly characterized.

We previously established that progranulin is not expressed in androgen-responsive LNCaP prostate cancer cells but instead acts as an autocrine growth factor for DU145 cells and promotes castration-resistant prostate cancer cell motility, invasion, and anchorage-independent growth (38). This study supported the hypothesis that progranulin was not important for prostate cancer initiation but might play an important role in prostate cancer progression and CRPC.

Sortilin, a single-pass type I transmembrane protein of the Vps10 family, binds progranulin in neurons and mediates progranulin targeting for lysosomal degradation (51). However, the role of sortilin in cancer is still very poorly characterized. In addition, whether sortilin is expressed in prostate cancer cells and plays any role in regulating progranulin action in prostate cancer has not previously established. Androgen-responsive LNCaP cells express very low levels of endogenous progranulin, do not respond to exogenous recombinant progranulin and do not express sortilin (52), suggesting that the progranulin/sortilin functional interaction may have a prevalent role in castration-resistant prostate cancer. Accordingly, in castration-resistant DU145 and PC3 cells, we detected very low levels of sortilin, which was associated with high levels of progranulin production and enhanced motility and invasion (52). Restoring sortilin expression in PC3 and DU145 cells decreased progranulin levels and inhibited motility, invasion, and anchorage-independent growth (52). These results were recapitulated by depleting endogenous progranulin in PC3 and DU145 cells. On the contrary, targeting endogenous sortilin by shRNA approaches enhanced progranulin levels and sustained motility, invasion, and anchorage-independent growth (52).

Similar to OC, Zheng et al. (53) reported that SLPI is overexpressed in CRPC patients and supports CRPC cell growth and invasion by functionally interacting with progranulin (53). SLPI overexpression in prostate cancer cells provides a proliferative advantage after castration by its anti-protease activity toward elastase and protecting progranulin from elastase-dependent degradation (53).

**Figure 2:**
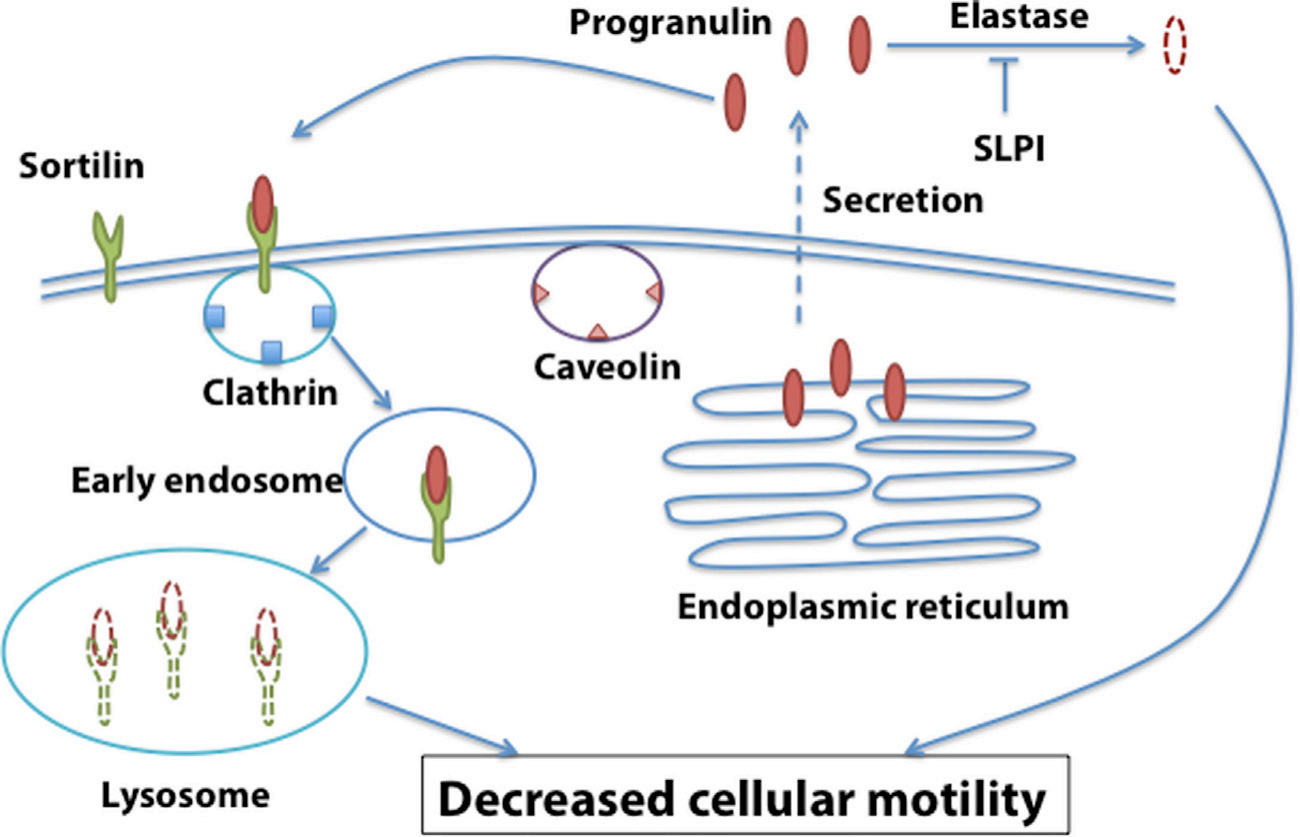
**Sortilin-dependent regulation of progranulin action in prostate cancer**. Sortilin promotes progranulin uptake through clathrin-dependent endocytosis and targets progranulin for degradation in the lysosomes. Sortilin loss is associated with enhanced progranulin levels and action in castration-resistant prostate cancer cells.

These results suggest that the functional interaction between sortilin, SLPI, and progranulin levels may be critical for prostate cancer, and sortilin/SLPI loss may contribute to progranulin-dependent action in prostate cancer progression (Figure 2).

The role of progranulin as a prostate cancer biomarker has not been clearly defined, and the data are also partially discordant. We investigated progranulin expression levels in available prostate cancer microarray studies using the ONCOMINE database (42). We found that progranulin mRNA expression was significantly increased in six primary prostate cancer data sets compared to controls (54–59). Additionally, primary tumors that have been treated with hormone therapy showed decreased progranulin mRNA levels (57). However, three studies showed contradictory results demonstrating that metastatic cancers had decreased progranulin expression levels compared to non-metastatic prostate cancers (54, 55, 60).

We evaluated by immunohistochemical analysis progranulin expression levels in normal versus prostate cancer sections and demonstrated that progranulin was expressed at low levels, in normal prostate but was instead upregulated in tumor glands (42), confirming the data by Pan et al. (61), who assessed progranulin (GP88) in normal prostate, prostatic intraepithelial neoplasia, and prostatic adenocarcinoma tissues. However, due to the limited sample size of both studies, additional analyses are indeed required to clearly define progranulin as a biomarker for prostate cancer progression.

## Conclusion

A vast body of evidence has emerged in support of the critical role of progranulin in promoting transformation of several cancer models. However, the mechanisms of progranulin action are still poorly characterized. Experiments are currently under way to identify novel regulators of progranulin signaling, which may constitute novel targets for therapy in tumors where progranulin plays a role.

## Author Contributions

RT, KL, AB, RVI, and AM wrote the paper and designed experiments. RT and KL performed experiments and discussed data. SB and S-QX performed experiments and discussed data.

## Conflict of Interest Statement

The authors declare that the research was conducted in the absence of any commercial or financial relationships that could be construed as a potential conflict of interest. The reviewer MD and handling editor declared their shared affiliation, and the handling editor states that the process nevertheless met the standards of a fair and objective review.
